# The Book World of Medicine and Science

**Published:** 1897-01-16

**Authors:** 


### THE BOOK WORLD OF MEDICINE AND SCIENCE.

A SYSTEM OF GYNÆCOLOGY, BY MANY WRITERS. Edited by T. CLIFFORD ALLBUTT, M.D., F.R.C.P., F.R.S., and W. S. PLAYFAIR, M.D., F.R.C.P. (London: Macmillan and Co. 1896. Price 25s. net.)

The exact relation of this volume to Allbutt's "System of Medicine" may not be easy to define, but at least it may be said that it is published on a plan so uniform in every way with the volumes of that work that it may usefully be accepted as one of the series. A careful perusal of its pages makes us feel that it worthily sustains the position of English gynæcology. The authors have been, for the most part, well chosen, the articles have evidently been very carefully prepared, and the whole work speaks with the voice of authority. We naturally turn first to the article contributed by the editor, Dr. Playfair, which is on "The Nervous System in Relation to Gynæcology." In dealing with this matter he begins at the beginning and speaks of the education of girls. In regard to the high schools and the more advanced colleges he says, "The one great fault of those who manage these educational establishments is that they have too often started on the absolutely untenable theory that the sexual factor is of secondary importance; and that there is little, if any, real distinction between a girl between the ages of 14 to 20 and a boy of the same age. I know of no large school for girls where the absolute distinction which exists between boys and girls, as regards the dominant menstrual function, is systematically cared for and attended to. The feeling of all school-mistresses seems to be antagonistic to such an admission." Dr. Playfair divides the cases of functional neuroses which are met with in practice into two classes. In one there may be some definite uterine or pelvic lesion, which is the starting point of reflex neurotic complications, and in these cases attention must be paid to the cure of the originating local complaint; in the other, some local lesion of minor importance may be found which may either be the secondary result of the general neurotic condition which is the predominant factor in the patient's health, or, although it may have once started the illness, may have become so overshadowed by its own secondary consequences that it has ceased to demand special attention, and, in fact, is best let alone. Dr. Playfair says, "Nothing can be more deplorably bad for a nervous emotional woman, whose general health is at a low ebb, than to have her attention constantly directed to her reproductive organs by vaginal examinations, repeated two or three times a week, pessaries constantly introduced for 'a slight displacement,' the cervix frequently cauterised, or the endometrium curetted, and the like; and yet these are things one incessantly sees in cases in which, on examination, no definite reason for such interference is found to exist." He insists on the presence of one condition in all cases of neurasthenia, one, namely, of defective general nutrition. This is the keynote to the treatment of a large number of cases of ill-health in women, which, although associated with some morbid condition referable to the reproductive organs, are quite incurable until the nutrition is improved.

The chapter on gynæcological therapeutics is good so far as the text is concerned. It seems a pity, however, that it should have been defaced by so many illustrations. The temptation offered by the surgical instrument maker's catalogue has been more than the author could resist, and as a result the pages are decorated with pictures of the most elementary instruments, such as a douche, a steriliser, a bed-bath, and two illustrations of Leiter's coils! The same criticism may also be applied to the article on electrical treatment, into which many useless woodcuts are introduced. This latter article is one of the very few which are not altogether satisfactory. If electrical treatment is to be used, no doubt the directions given are proper enough; but they seem curiously empirical, and in giving the reasons for his faith it cannot be said that Dr. Milne Murray is very happy. A very good article by Dr. Herman treats of the diseases arising from mechanical injury during child-birth. The article by Mr. Bland Sutton on "Extra Uterine Gestation" is one which will be read with great interest. It is both well written and well thought out, and expresses the views of the author with great clearness. In this article we see an example of the proper use of woodcuts. They are all aptly illustrative of points raised in the text, and they are all extremely well drawn and engraved. Dr. Cullingworth contributes a useful and practical article on "Pelvic Inflammation," a subject which has given rise to much disputation in times past. He maintains the uniformly septic origin of pelvic cellulitis and the equally uniform dependence of pelvic peritonitis on some pre-existing disease within the pelvis. Stress is laid, and rightly laid, on the fact that in the large majority of non-puerperal cases, pelvic peritonitis is due to gonorrhœal salpingitis, and that, lightly as gonorrhœa is looked upon in this country, it probably destroys the health of a larger number of women than does even the much more dreaded poison of syphilis. The article on "Pelvic Hæmatocele" we cannot look upon as altogether satisfactory. We might go even further, for it is difficult to avoid the conclusion that the careful description of the many possible sources of the blood in pelvic hæmotocele tends to obscure the preponderating importance of the one great cause of the condition, and may thus lead to hesitation in one of the gravest emergencies which can arise. The articles on the more purely operative part of gynæcology are thoroughly practical. Hysterectomy is dealt with by Mr. Knowsley Thornton; Plastic Operations by Dr. John Phillips, whose careful diagrams are very useful in elucidating his remarks; and Ovariotomy by Mr. Greig Smith, who, as is his wont, appeals to personal experience and practical knowledge in all that he lays down. We feel sure that this System will for some time to come remain the standard book of reference on the subjects on which it treats.

